# Effects of Chemically and Green Synthesized Zinc Oxide Nanoparticles on Shelf Life and Sensory Quality of Minced Fish (*Pangasius hypophthalmus*)

**DOI:** 10.3390/foods13172810

**Published:** 2024-09-04

**Authors:** Achinta Mahato, Paresh Nath Chatterjee, Sougata Sarkar, Arup Ratan Sen, Aruna Pal, Sovan Roy, Amlan Kumar Patra

**Affiliations:** 1 Department of Animal Nutrition, West Bengal University of Animal and Fishery Sciences, Kolkata 730037, India; achinta1991@gmail.com; 2 Department of Fish Nutrition, West Bengal University of Animal and Fishery Sciences, Kolkata 700094, India; 3 Ramakrishna Mission Vivekananda Centenary College, Rahara, Khardaha 700118, India; sougata.sarkar81@gmail.com; 4 ICAR-Central Institute of Fisheries Education, Kolkata 700091, India; senarup68@gmail.com; 5 Department of Livestock Farm Complex, West Bengal University of Animal and Fishery Sciences, Kolkata 700037, India; arunachatterjee@gmail.com; 6 West Bengal State Council of Science and Technology, Department of Science & Technology and Biotechnology, Vigyan Chetna Bhavan, Salt Lake, Kolkata 700064, India; sovanroy@gmail.com; 7 American Institute for Goat Research, Langston University, Langston, OK 73050, USA

**Keywords:** nanoparticle, zinc, shelf life, fish, fortification

## Abstract

The purpose of this study was to investigate the effect of chemically and green synthesized zinc oxide nanoparticles (ZnO-NPs) on the shelf life and sensory quality of fish meat. In this study, ZnO-NPs were synthesized by employing the colloidal chemistry (CZnO-NPs) and green synthesis (GZnO-NPs) methods, and they were also characterized to assess their morphology. The synthesized ZnO-NPs, ZnO, and zinc acetate (ZnA) were used for the preservation and fortification of fish (*Pangasius hypophthalmus*) meat at 20 mg/kg of Zn. In a six-day storage study at 4 °C, the fish samples were evaluated for their sensory attributes (color and odor), physicochemical quality (pH and total volatile base nitrogen), oxidative changes (thiobarbituric acid-reactive substances and peroxide value), and microbial loads at 0, 3, and 6 days of storage. The fortification of raw fish with the synthesized CZnO-NPs produced better sensory attributes (color and odor) and maintained a pH non-conducive to microbial growth throughout the entire storage period compared with the control, ZnO, and ZnA-fortified samples. The GZnO-NPs largely did not provide any added advantage over CZnO-NPs but sometimes responded better than the control, ZnO, and ZnA samples. Oxidative status and total volatile base nitrogen were lower for CZnO-NPs in refrigerated fish compared with the other treatments. The ZnO-NP-fortified fish had the lowest counts of total viable bacteria, coliforms, *Staphylococcus* spp., and *Vibrio* spp. Hence, the fortification of fish with synthesized CZnO-NPs is promising as a food additive to reduce microbial spoilage and lipid peroxidation of fish in storage.

## 1. Introduction 

Fish foods are very delicious and are consumed throughout the world as a basic protein source containing health beneficial fatty acids providing nutritional food security [1]. The storage of fish remains a major concern as it often leads to quality deterioration due to lipid oxidation, protein denaturation and microbial growth, and organoleptic trait alterations [2,3]. Fish is highly prone to oxidation and spoils easily, giving off odor due to incorrect storage methods, and it is mostly preserved by refrigeration, chilling, pressure treatment, and irradiation [4]. Additionally, many synthetic and natural food additives are used to augment the shelf life of fish, with variable results [2]. Zinc oxide (ZnO) exerts antimicrobial activities against both Gram-positive and Gram-negative bacteria [5]. With the advent of nanobiotechnology, the inherent antimicrobial efficacy of inorganic minerals could be magnified many fold by transforming it into nanoforms, which could be useful for food packaging and preservation [6,7,8,9]. The antibacterial and antifungal actions of ZnO are also enhanced by reducing its size to the nanoscale [5,10,11]. Many nanominerals are potent antimicrobial agents, including ZnO nanoparticles (ZnO-NPs), which can also be exploited as food preservatives [12,13]. Due to the potent antimicrobial efficacy of ZnO-NPs, their direct addition to foods is of recent interest in terms of reducing microbial contaminants and extending shelf life [14,15,16]. ZnO-NPs are an emerging additive for bio-nanocomposite coatings or films for food packaging with highly potent antimicrobial properties against food spoilage microbes without affecting organoleptic traits and food quality [17,18,19,20,21]. The chemically synthesized ZnO-NPs added in media showed strong antibacterial action against various foodborne pathogens compared with the bulk ZnO or control [22,23]. The combination of ZnO-NPs with other natural or synthetic antimicrobial compounds has been demonstrated to be very promising in food preservation and the enhancement of shelf life [24]. The shelf life of rainbow trout filets was further enhanced by fortifying ZnO-NPs with *Mentha spicata* essential oil [25]. The US Food and Drug Administration has listed ZnO as GRAS (generally recognized as safe) [26]. Nanostructures of ZnO, being chemically stable and harmless [27], can be used in food preservation practices due to their antimicrobial efficacy [28].

Microencapsulated Zn compounds have been prepared for Zn fortification in foods and the improvement of bioavailability [29]. Nanoscale ZnO particles can be exploited, which, in turn, not only enhance shelf life but also enrich the meat with the addition of Zn to human diets, which is an essential mineral with numerous physiological roles in the body, including enzyme activities; DNA replication; protein, carbohydrate, and nucleic acid metabolism; cell division; hormone secretion; and immunity [30,31]. Diets in many developing countries, including India, are deficient in Zn; hence, this fortified fish would be a good source of Zn supplementation. Among trace minerals, Zn is the second most abundant in the animal body, but it cannot be stored in sufficient amounts in the body, and thus the body requires regular dietary Zn intake to meet its physiological needs.

Absorption of Zn is highly challenging and differs in humans and animals with age and with the site of the gastro-intestinal tract. Zinc requirement ranges between 20 and 80 ppm in different classes of livestock and 8 mg/day to 11 mg/day in adult humans, and it is absorbed throughout the small intestine as Zn ions, as a complex with amino acids/chelates. The bioavailability of inorganic sources of Zn is very poor [32]. Organic sources of zinc have been studied by several researchers [33,34], but they have not been adopted extensively. More recently, it has been postulated that nanodimensional mineral particles are believed to be absorbed and transported via an altered route, resulting in the enhanced bioavailability of these nanominerals [35,36,37]. The high bioavailability of different nanomineral particles is assumed to be due to some of their unique properties including high catalytic efficiency, greater surface area and activity, and stronger adsorbing capability [9]. Due to these characteristics, nanoparticles are used in drug design, targeting therapeutic agents to specific organs [38]. As pointed out above, ZnO-NPs present in nanocomposite films or added in media have been evaluated for their antimicrobial properties in many studies, but the comparative effects of chemically or green synthesized ZnO-NPs directly added to fish filets for Zn fortification for shelf life enhancement and sensory quality are not available [39]. Due to the enhanced antimicrobial effects and greater bioavailability of ZnO-NPs and the necessity of Zn for the fortification of fish meat, this study aimed to synthesize ZnO-NPs by chemical and green synthesis methods and the evaluation of their preservation and fortification in terms of the shelf life and sensory quality of fish meat.

## 2. Materials and Methods

### 2.1. Chemical Synthesis of ZnO Nanoparticles

An environmentally benign wet chemical method was applied for the synthesis of ZnO-NPs following the procedure of Wang et al. [40] with some modifications. In brief, an aqueous solution (10 mL) of a cationic surfactant, cetyltrimethyl ammonium bromide (12 mM) was added dropwise to an aqueous solution (25 mL) of zinc acetate (6 mM) under vigorous stirring for 30 min. Subsequently, 4 mL ammonia solution (28 to 30%) was added dropwise to this solution, and the solution was continuously stirred for another 15 min. Finally, the solution mixture was transferred into stoppered glass tubes (10 mL capacity) and was heated in an oven at 100 °C for 24 h. After cooling to room temperature, the solution was centrifuged. The white precipitate obtained was washed with water and finally with ethanol repetitively by centrifugation to remove the soluble impurities. Then, it was dried for 2 h at 60 °C in a hot air oven. After drying, the white soft powder of ZnO-NPs was preserved for characterizations and further use.

### 2.2. Green Synthesis of ZnO Nanoparticles

Aqueous extract of guava (*Psidium guajava*) leaves was utilized as a reducing agent as well as a stabilizing agent for the synthesis of ZnO-NPs following a green method. Green synthesis of ZnO-NPs was followed as per Elumalai and Velmurugan [41]. Fresh guava leaves (50 g) were washed several times with distilled water and cut into small pieces. They were dried under shade and then boiled with 150 mL deionised water for 30 min. After cooling, the mixture was filtered through the millipore filter paper. In a clean beaker, 45 mL of guava leaf extract was taken, and 1.5 g of zinc acetate was added to it with vigorous magnetic stirring and kept for 1 h at boiling temperature. Then, the beaker was kept overnight in a hot air oven at 100–105 °C. The brownish residue was ground with pestle and mortar, washed several times with distilled water followed by ethanol, and then centrifuged at 5000 rpm for 10 min. The particles were then spread out as a thin film in Petri dishes for drying at 70 °C for 2 h. After drying, brownish soft powder was stored for further analysis and investigation of the shelf life of fish.

### 2.3. Characterization of ZnO Nanoparticles

The size and shape of the synthesized ZnO-NPs were characterized by field-emission scanning electron microscopy (FESEM; JSM 7600F, JEOL, Tokyo, Japan). Elemental composition analysis of ZnO-NPs was performed by energy-dispersive X-ray (EDX) (SUPRA 55 VP 4132 Carl Zeiss, Oberkochen, Germany) analysis. Powder X-ray diffraction (XRD) (Bruker D8 Advance, Karlsruhe, Germany) was performed for identification, purity and quantitative analysis of ZnO-NPs in range from 30° to 80° Cu *K*α radiations (*k* = 0.15406 nm). The FESEM analysis was carried out at Nano Science Centre, Calcutta University, India, whereas EDX and powder XRD analyses were performed at Central Instrumentation Facility, Kalyani University, India.

### 2.4. Preservation of Fish

In an initial trial, graded concentrations of ZnO-NPs were used and the desired concentration for fortification was determined based on the physicochemical characteristics of the meat. Then, a comparative storage study of minced fish was carried out to evaluate the efficacy of ZnO-NPs as compared to its inorganic counterparts, i.e., ZnO and ZnA, to evaluate nano size versus larger sizes of these chemicals. Fresh pangasius (*Pangasius hypophthalmus*) fish (average weight of 800 to 900 g) procured from a fish market (Belgachia, Kolkata, India) were beheaded, de-scaled, filleted and skinned manually. In recent years, this fish has attracted increased attention in India due to consumer demand and the higher rate at which they gain weight. The fillets were separated and minced using a mincer (Talleres Ramon, Spain, Barcelona; Model No. P-32) with an 8 mm plate under good hygienic and sanitary conditions. Initially, the synthesized ZnO-NPs were mixed with minced fish samples in triplicates to standardize the concentration of ZnO-NPs with graded levels of addition (0, 5, 10, 20 and 40 mg/kg of fresh fish) for the appearance of aesthetic appeal of fish samples. The best sensory results were found at 20 mg/kg in an 8-day storage study (Supplementary Figure S1). Thereafter, a dose of 20 mg/kg was used for a 6-day-long comparative storage study of minced fish at 4 °C with different sources of Zn, which were (1) control (without any added Zn), (2) fish samples fortified with 20 mg/kg of inorganic ZnO (ZnO), (3) fish samples fortified with 20 mg/kg of chemically synthesized ZnO-NPs (CZnO-NPs), (4) fish samples fortified with 20 mg/kg of inorganic zinc acetate (ZnA), and (5) fish samples fortified with 20 mg/kg of green synthesized ZnO-NPs (GZnO-NPs). Each treatment had three replicates for day 0, 3, and 6, and the minced fish fortified with different sources of Zn were mixed properly, packed in laminated pouches, and kept for 6 days in a refrigerator (4 ± 1 °C) for the sensory, physicochemical, and microbial studies of storage stability. The whole set of the experiment was repeated four times and completed within two months.

### 2.5. Chemical and Microbiological Analysis

#### 2.5.1. Measurement of pH

The pH of the treated fish samples was determined on day 0, 3, and 6 by homogenizing 10 g of sample with 50 mL distilled water using a tissue homogenizer (WiseMix, HG-15D; Daihan Scientifics, Wonju-si, Gangwon-do, Republic of Korea) for 1 min. The pH of the suspension was recorded using a digital pH meter (Thermo Scientific Orion Star A111, Hudson, MA, USA), which was calibrated against a buffer of pH 4, 7, and 10. The pH values of each sample were determined in triplicates.

#### 2.5.2. Thiobarbituric Acid Reactive Substances

The thiobarbituric acid reactive substance (TBARS) value was measured following the procedure of Witte et al. [42]. Briefly, trichloroacetic acid (TCA) extracts of the minced fish samples were prepared by homogenizing 4 g of fish sample with 20 mL of cooled TCA solution (20 g/100 mL) for 2 min in an ultra-turrax homogenizer. The contents were allowed to stay for 10 min for better extraction and subsequently were centrifuged at 3000 rpm (CPR-24; Remi Instruments, Mumbai, India) for 10 min.

The supernatant (3 mL) was mixed with an equal volume of thiobarbituric acid (1 g/L; TBA) solution. For the blank, the same procedure was followed as described above except that 3 mL of cooled TCA solution (200 g/L) was added instead of the TCA extract. The mixture was boiled in a water bath for 30 min and then cooled to room temperature, and absorbance was measured at 530 nm using an UV–Vis spectrophotometer (Thermo Electron Corporation, Cambridge, UK; Model No. AQA 133204). TBARS values were calculated by extrapolating from a standard curve and were expressed in mg malondialdehyde/kg fish meat.

#### 2.5.3. Peroxide Value

The peroxide value (PV) was determined following the method of Jacobs [43]. Fish samples (10 g) were homogenized after adding anhydrous sodium sulfate (15 g) to eliminate moisture. Fat was extracted with chloroform (50 mL) and the filtrate was collected. The chloroform filtrate (15 mL) was mixed with glacial acetic acid (15 mL) and potassium iodide solution (100 g/L; 10 mL). The mixture was then kept in a dark place for 10 min and distilled water (50 mL) was added to it. To measure the released iodide, the content was titrated with sodium thiosulfate solution (0.02 N) in the presence of freshly prepared starch solution (10 g/L; 1 mL) until the disappearance of the blue color. The PV was calculated and expressed as meq of active O_2_/kg of fat.

#### 2.5.4. Total Volatile Base Nitrogen

The content of total volatile base nitrogen (TVB-N) in fish samples was determined following the Conway microdiffusion method [44]. Fish samples (10 g) were homogenized after adding anhydrous sodium sulfate (15 g) to eliminate moisture. The minced fish sample (100 g) was homogenized with 5 g TCA in a porcelain basin to make a fine slurry. After 30 min, it was filtered through a number 5 Whatman paper, and the filtrate was used for the assay. The filtrate (1 mL) was treated with 1 mL of saturated potassium carbonate solution, and the volatile base was diffused over into 2 mL boric acid solution (10 g/L) in a Conway microdiffusion unit. It was incubated for 3 h at 37 °C with intermittent shaking. The boric acid solution was titrated with sulfuric acid solution (0.02 N), and the TVB-N (mg N/100 mL of filtrate) was calculated as follows:

TVB-N (mg/100 g fish) = reading of the burette × 0.02 × 14 × 100

#### 2.5.5. Microbiological Analysis

The microbial analysis of treated fish samples was performed on day 0, 3, and 6 as per the methods described by APHA [45]. The ready-made media (Hi-media Laboratories (P) Ltd., Mumbai, India) were utilized for the enumeration of different microbes. Serial dilutions of minced fish were prepared using sterile peptone water (1 g/kg) as a diluent. For the total viable bacterial counts, Plate Count Agar (M091) medium was prepared in Petri dish plates in duplicate sets. For the coliform (Violet Red Bile Agar M049A), *Vibrio* spp. (Vibrio Agar M820), and *Staphylococcus* spp. (Mannitol Salt Agar M118) bacterial counts, specific media were prepared in duplicate Petri dish plates. All plates were inoculated with 1 mL aliquots from suitable dilutions and incubated at 37 ± 1 °C for 48 h. The colonies were counted, and the counts were expressed as colony forming units (CFUs) per gram of sample. The numbers of colonies were multiplied with reciprocal of the dilution and expressed as log_10_CFU/g of fish samples.

#### 2.5.6. Sensory Evaluation

The minced fish samples were evaluated by 10 trained panelists for color/appearance and odor using a 5-point descriptive scale. The sensory attributes and their descriptions were adopted from Camo et al. [46] with slight modifications, i.e., for color (from 5—extremely desirable to 1—undesirable) and for odor (from 5—excellent to 1—poor). All analyses were performed on day 0, 3, and 6 of storage in triplicates.

### 2.6. Statistical Analysis 

The experiment was conducted in triplicates using four different lots of minced fish samples. The data analysis was performed using SPSS 16 for Windows, using a one-way analysis of variance to determine the effects of the treatment and storage period. The results were expressed as mean (±standard deviation) and pooled standard error of mean. When the treatment or storage period effects were significant (*p* < 0.05), means were compared using Duncan’s multiple-range tests considering the significance level at *p* < 0.05 for all experimental data. Pearson correlation (*r*) among the variables evaluated in this study was assessed using SPSS.

## 3. Results and Discussion

### 3.1. Field-Emission Scanning Electron Microscopy

**Figure 1:**
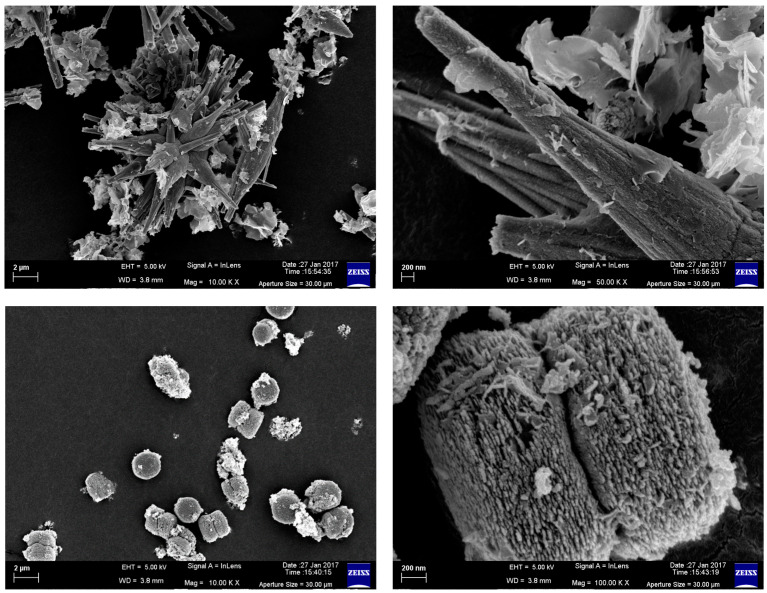
Field-emission scanning electron microscopy image of chemically synthesized ZnO nanoparticles (upper panel) and green synthesized ZnO nanoparticles.

The morphologies of the synthesized ZnO nanostructures were examined through FESEM. The FESEM image of the synthesized ZnO nanostructures prepared at a molar ratio of [Zn(OAc)_2_]/[NH_3_]/[CTAB] = 1:7:2 at a solution pH of ~10 is shown in Figure 1. The reaction involved the hydrolysis of the ZnA under a basic condition achieved by the addition of ammonia. The FESEM image of CZnO-NPs clearly depicts the formation of well-defined ZnO nanostructures with a spindle-like morphology. We can observe from the FESEM images that the growth occurred in a bundled fashion. The bundled growth of ZnO-NPs is associated with the concentrically grown individual nanospindle. The spindles were of micrometer length but a nanoscale diameter (10–13 μm with a width of 500–700 nm). We can also observe that these nanobundles remained associated with flower-like morphology but in minor proportions. The enlarged view shows that the surface of each of the spindles was rough in nature and formed through the continuous aggregation of ZnO-NPs. Here, it is important to note that the cationic surfactant, CTAB, plays a major role in controlling the shape as well as the one-dimensional form of the nanostructures.

### 3.2. Elemental Analysis

**Figure 2:**
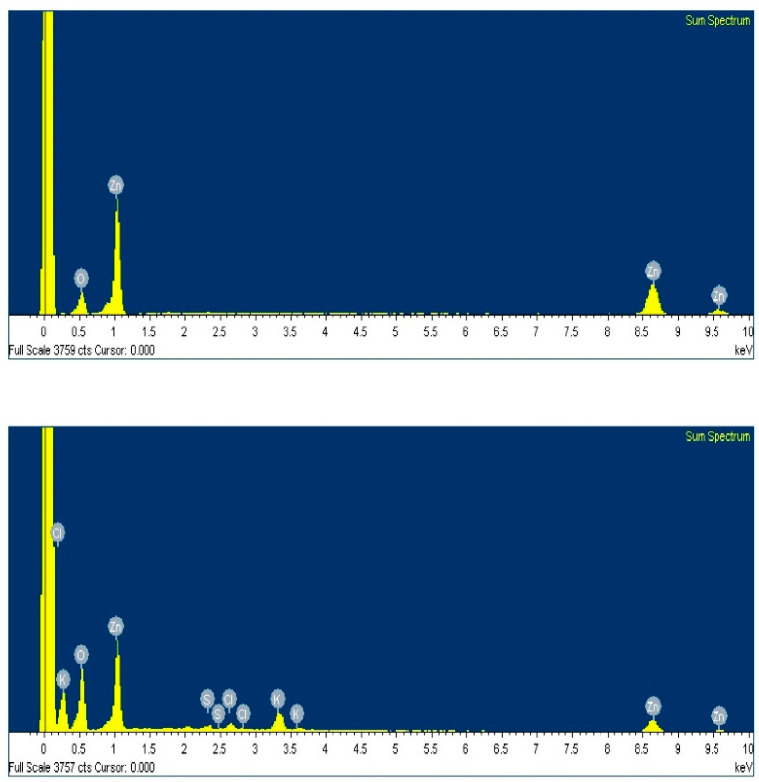
Energy-dispersive X-ray analysis of chemically synthesized ZnO (upper panel) and green synthesized ZnO (lower panel) nanoparticles.

The elemental analysis revealed the presence of both Zn and O in the case of CZnO-NPs (both the peaks for Zn and O were intense), thereby substantiating the compositional purity of the synthesized CZnO-NPs (Figure 2). The ZnO-NPs synthesized using the guava leaf extract also revealed the presence of Zn along with Cl, K, and S. The intensified peaks of Zn confirmed the high Zn content within the synthesized material. The synthesized CZnO-NPs and GZnO-NPs were found to contain 80.45% and 32.34% Zn, respectively.

### 3.3. Powder X-ray Diffraction

**Figure 3:**
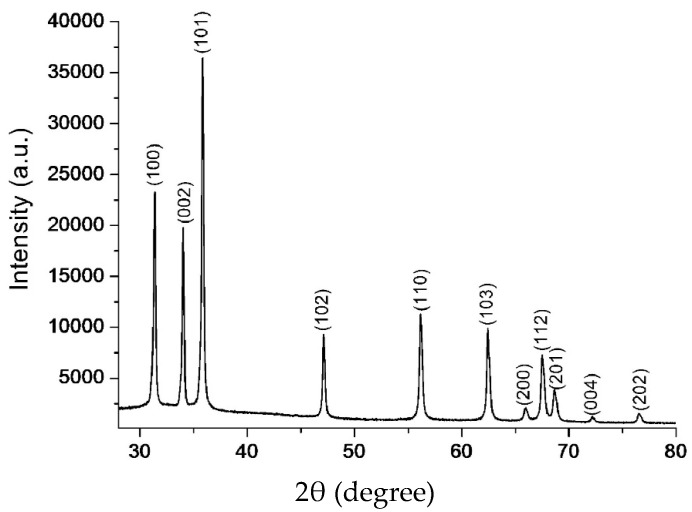
Powder X-ray diffraction analysis of chemically synthesized ZnO nanoparticles.

The powder XRD pattern of the CZnO-NPs was analyzed as it resulted in better effects based on the different tested variables in minced fish samples, as presented in Figure 3. All the peaks could be clearly assigned to the pure hexagonal Wurzite structure of ZnO, which could also be matched with the reported data (JCPDS number: 79–0206). Here, it is worth mentioning that the pattern was devoid of any other peaks, which clearly indicated that the sample was pure without any other contaminants.

### 3.4. Physicochemical Quality

#### 3.4.1. pH of Fish on Storage

The pH of the fortified meat samples remained similar (*p* > 0.05) among the different treatments on day 0 of storage (Table 1). On day 3, fortification of fish with ZnO-NPs, ZnO, and ZnA resulted in lower pH compared with the control, and the lowest pH was observed for CZnO-NPs. Fortification of CZnO-NPs proved to be beneficial for the fish stored for a longer period, as observed in the present experiment compared with other treatments on day 6. The lower the pH of fish, the lower the chance of microbial growth. Addition of GZnO-NPs might be recommended up to the third day of storage. The control fish samples with a higher pH value throughout the study are supposed to be more prone for microbial growth. Mixing fish with inorganic ZnA or ZnO might be beneficial up to the third day of storage compared with the control fish. With the advancement of storage days, the pH of all the treated fish samples significantly (*p* < 0.05) increased, except for CZnO-NPs, which was likely due to production of alkaline compounds such as tri-methyl amines and ammonia during bacterial growth, suggesting deterioration of the quality of fish [47,48,49]. In the present study, CZnO-NPs appear to be a promising additive increasing the value of fish.

#### 3.4.2. Thiobarbituric Acid

The influence of fortification of different Zn sources on TBARS content in minced fish during the refrigerated storage study revealed that CZnO-NPs provided the best defense against oxidative damage during prolonged storage, followed by ZnO. The antioxidant property of ZnO might be due to electron donation capability of oxygen atoms in ZnO-NPs [50]. Premixing with inorganic-Zn (ZnO and ZnA) and GZnO-NPs had no influence (*p* > 0.05) during the initial phase of storage (third day), but it might be protective over a longer duration of storage (i.e., sixth day onward) compared with the untreated control (Table 1). With the increasing time of storage, TBARS values increased in all the treated and untreated meat samples. Similar trends were reported when rainbow trout fillets were treated with essential oils (clove versus thyme) loaded in gum-based coatings [51]. In another study, the beneficial role of fortification was established, as cooked pork sausages exhibited the slowest change in TBARS value when treated with a mixture of chitosan (20 g/kg) and clove oil (15 mL/kg) [52]. Minced beef meat treated with a polylactic acid film containing *Ziziphora clinopodioides* essential oil (0, 10, and 20 mL/L) alone and in combination with different concentrations of a propolis ethanolic extract (0, 10, and 20 mL/L) and cellulose nanoparticles (0 and 10 g/L) also exhibited similar trends in TBARS changes, with the best results reported with the higher combination ratios [53]. The antioxidant activity of ZnO has also been reported in several in vitro and in vivo studies [50,54,55,56].

#### 3.4.3. Peroxide Value

Fortification with different Zn sources did not influence (*p* > 0.05) the PV of minced fish on the initial day (day 0) of storage and the final day of storage (sixth day). Fish samples fortified with ZnO-NPs and GZnO-NPs exhibited minimum oxidative changes compared with other treatments on day 3 (Table 1). Inclusion of inorganic Zn (ZnO and ZnA) as an additive to fish samples exerted some degree of protection up to the third day of storage. Lowered PV values seem to be associated with the better aesthetic appeal of the treated fish compared with the untreated control during long-term storage. In the untreated samples (control), higher lipid oxidation is often associated with the increased microbial lipase and phospholipase activity, which in turn increases the free fatty acids that are sensitive to oxidation [57]. In a study, the PV values of stored rainbow trout (refrigerated storage) were lower when it was coated with chitosan (2%) or enriched with cinnamon oil (1.5%), and the stored meat that received a combined treatment (i.e., chitosan + cinnamon oil) had the lowest PV value [58]. Mohebi and Shahbazi [57] tested the effect of gelatin and chitosan films containing cellulose nanoparticles (0 and 1%), *Ziziphora clinopodioides* essential oil (0 and 1%), and pomegranate peel extract (0 and 1%), separately and in combinations, on the shelf life of fresh shrimp during refrigerated storage; the chitosan films treated with all three additives resulted in the lowest PV scores.

#### 3.4.4. Total Volatile Base Nitrogen

The influence of fortification of different Zn sources on the TVB-N value of fish at different storage periods is presented in Table 1. No difference was observed on the first day of storage. On day 3, CZnO-NPs fortification performed the best among all the additives, followed by ZnO and GznO-NPs, and then ZnA. On day 6, the CZnO-NPs-fortification performed better than the GZnO-NPs. The unfortified control meat remained the most inferior in the present storage study with the highest TVB-N value throughout the storage periods. The TVB-N value is mainly a combination of trimethylamine, dimethylamine, and ammonia, which is considered an important quality control indicator for assessing the freshness of fish and meat [59]. The TVB-N values increased with the storage time due to oxidative deamination of amino acids caused by increased bacterial populations [60] and activity of endogenous enzymes [48,61]. The present findings agreed with the earlier report of Jouki et al. [59] who studied the effect of quince seed mucilage films containing different concentrations of oregano and thyme essential oils (1, 1.5 and 2%), separately and in combination, on the extension of the shelf life of rainbow trout fillets. They observed that combined treatments remained superior in terms of lowered TVB-N values in the meat samples upon prolonged storage. Similarly, the effect of chitosan and gelatin films incorporated with cellulose nanoparticles (0 and 1%), grape seed extract (1 and 2%), and *Z. clinopodioides* essential oil (1 and 2%), separately and in combination, on the shelf life of minced trout fillet was studied by Kakaei and Shahbazi [61], where better TVB-N results were reported for fillets treated with both *Z. clinopodioides* essential oil and grape seed extract. The efficacy of fortification with ZnO-NPs in delaying the deterioration of meat under refrigerated storage has recently been established [25], where stored rainbow trout fillet samples had a significantly lowered TVB-N values, even after 14 days of storage. This decreased TVB-N value might be associated with the lowered incidence of pathogenic loads in the stored meat samples, which was observed in the present study.

### 3.5. Microbial Analysis of Fortified Fish

Microbial loads of the fortified fish at different days of refrigerated storage showed that CZnO-NPs-fortified fish had the lowest (*p* < 0.001) total viable count throughout the storage period (Table 2). Fortification of fish with GZnO-NPs showed its potential until the third day of refrigerated storage, but no added effect was observed thereafter in comparison with the ZnO and ZnA. ZnO has potent antimicrobial activity, and several ZnO preparations are used for the healing of various wounds [62]. It has been suggested that ZnO releases reactive oxygen species (ROS) which, together with Zn ions, likely attack the negatively charged cell wall and thus impair the bacterial cell wall synthesis process [63]. Total viable bacterial counts increased in all treatment groups with the increasing duration of fish storage.

The antimicrobial potential of ZnO-NPs against other common microbial contaminants (coliforms, *Vibrio* spp., and *Staphylococcus* spp.) of fortified fish samples was also observed in the present investigation. On the first day of storage, the coliform number (log CFU/g) remained same irrespective of treatment groups (Table 2); meanwhile, with progressive days of storage, significant variations in their number were observed depending upon the antimicrobial efficacy of the fortification, i.e., the highest efficacy was shown by CznO-NPs, followed by GznO-NPs, and then ZnO and ZnA. On day 6, all the treatments behaved similarly, except for CZnO-NPs fortification, which showed greatest inhibitory effect on the coliforms. Though the microbial loads were below the threshold value of spoilage of 7 log10 CFU up to the end of the storage period, the greater antimicrobial nature of CZnO-NPs might be associated with their size and charge to surface ratio of the nanomolecules [12,13].

Significant decreases (*p* < 0.05) in *Vibrio* and *Staphylococci* counts were observed on day 3 and 6 of storage when the experimental minced fish samples were fortified with CZnO-NPs and GZnO-NPs compared with the control, ZnO, and ZnA; however, fortification with CZnO-NPs outperformed compared with their inorganic counterparts and GZnO-NPs. Fortifications with ZnO and ZnA were usually found to have comparable effects, but it lowered the *Vibrio* and *Staphylococci* counts compared with the control. The antibacterial properties of GZnO-NPs were usually similar to ZnO and ZnA, but lower than CZnO-NPs, indicating that green synthesis of ZnO-NPs using the guava leaf extract was not highly effective against pathogenic bacteria. The properties, morphology, and size of the ZnO-NPs vary depending upon the plant extracts used, reaction time, and temperature [64], which influence the extent to which the growth of different types of microbes is inhibited [65]. Considering its potent antimicrobial efficacy, direct addition of CZnO-NPs to foods is of recent interest with an aim to reduce the microbial contaminants and to extend the shelf life and food safety [16,66]. Disk diffusion and liquid media tests showed that films containing ZnO-NPs inhibited the growth of *Staphylococcus aureus*, *Salmonella* Typhimurium, and *Pseudomonas aeruginosa* [22]. The superiority of ZnO-NPs over other Zn preparations against a wide array of foodborne pathogens has been reported by Tayel et al. [67]. The shelf life of raw beef meat could also be extended by treating chitosan films incorporated with ZnO [20]. Storage of cod fillets in boxes containing ZnO-NPs as coatings resulted in reduced mesophilic and psychotropic bacterial numbers compared with the control or the coatings with polylysine after 3 days and 6 days of storage [19]. Marcous et al. [68] extensively studied the antibacterial effect of ZnO-NPs coated with low-density polyethylene films on *E. coli* in calf minced meat. When ZnO-NPs were combined with other natural or synthetic antimicrobials, they proved to be very promising in food preservation and enhancing shelf life [24]. The shelf life of rainbow trout fillets was further enhanced by fortifying with ZnO-NPs along with *Mentha spicata* essential oil [25]. Rezaei and Shahbazi [60] also recorded the inhibitory potential of ZnO in silver carp fillets coated with different essential oils. Considering their potential antimicrobial activity, different ZnO nanostructures are of recent interest for food packaging [69]. ZnO nanostructures, being chemically stable and harmless [27], can be strategically used in food preservation practices to exploit their antimicrobial efficacy [28].

### 3.6. Sensory Attributes of Fish in Storage

The color and odor of minced fish during refrigerated storage did not vary (*p* > 0.05) among the treatments until day 3 of storage (Table 3). On day 6 of storage, the best color appeal was observed in CZnO-NPs-fortified fish meat compared with the control and GZnO-NPs, but there were no differences among CZnO-NP, ZnO, and ZnA. Additionally, there were no differences among control, ZnA, and GZnO-NP for the color of minced fish. The odor was not different on day 0 and 3, but on day 6 of storage, odor was better for CZnO-NPs-fortified fish meat compared with the control and other treatments except ZnO. Inclusion of GZnO-NPs in the fish did not provide any added advantage for color and odor in the present experiment. Again, the quality (both color and odor) deteriorated (*p* < 0.05) in all the treated meat samples with the duration of storage. However, among all the treatments, CZnO-NPs-fortified meat samples were found to be aesthetically more appealing in terms of color and odor on day 6 of storage. The sensory score for color and odor of fish samples ranged from 2 to 3, indicating moderate desirability up to the end of the storage period.

In a previous study, fortification of silver carp fillets with ZnO at 5 g/kg proved to be beneficial, and a synergistic effect of ZnO was found when coated with *Ziziphora clinopodioides* essential oil at 5 g/kg and apple peel extract at 10 g/kg in a long-time storage study (Rezaei and Shahbazi, 2018). Shahbazi and Shavisi [25] also observed that during the storage of rainbow trout fillets at a higher level (40 g/kg) of ZnO fortification (along with chitosan and *Mentha spicata* essential oils) proved better in terms of higher sensory score of treated fillets compared with ZnO used at 20 g/kg fish fillet. The cod fillets stored in boxes containing biopolyethylene films coated with ZnO-NPs had the lowest gumminess compared with the control or the films coated with 2% polylysine, and these fillets had the best quality and freshness of cod fillets after 6 days of storage at 5 °C [19]. Broiler breast fillets dipped in sterile distilled water spoiled on day 3 of storage (4 °C), but the fillets dipped in suspension containing 5, 8 and 10 mM of ZnO-NPs (20 nm size) had acceptability until day 9 of storage, and even up to 12 days of storage at concentrations of 8 and 10 mM [70].

### 3.7. Correlations

Pearson correlation analysis showed that there were significant (*p* < 0.001) correlations among all the variables (Table 4). All correlations were positive, except for the correlations with color and odor that were negatively associated. This finding indicated that these variables used for evaluation of fish quality are involved with each other either positively or negatively, which was also reported in other studies with fish fillets during ice storage [71].

## 4. Conclusions

In the present set of experiments, the synthesis of nanodimensional ZnO particles through an environmentally friendly colloidal chemistry method was successful, as determined by the analysis of micro-analytical structures. Fortification of raw minced fish with the synthesized CZnO-NPs at 20 mg/kg revealed better sensory attributes (color and odor) and maintained a pH not conducive to microbial growth throughout the entire study. The nanostructured ZnO particles synthesized through the green route largely did not provide any added advantage over CZnO-NPs. The oxidative assays (TBARS and PV) as well as TVB-N values suggested the promising nature of CZnO-NPs as a preservative for refrigerated storage of fish. The ZnO-NP-fortified fish was also found to be the least prone to microbial spoilage, as observed in the lowest counts of total viable bacteria, coliforms, *Staphylococcus,* and *Vibrio*. Hence, the fortification of fish with synthesized CZnO-NPs is promising as a food preservative and should be further studied to exploit its potential benefits in human and animal biological systems.

## Figures and Tables

**Table 1:** Effect of different zinc sources on physicochemical quality (mean ± standard deviation) of minced fish during refrigerated storage (4 ± 1 °C).

Item	Treatment					SEM	*p* Value
	C	ZnO	CZnO-NP	ZnA	GZnO-NP		
pH							
Day 0	6.26 ± 0.017 ^B^	6.25 ± 0.022 ^C^	6.25 ± 0.021 ^B^	6.26 ± 0.011 ^C^	6.25 ± 0.014 ^C^	0.008	0.64
Day 3	6.74 ± 0.109 ^Ap^	6.38 ± 0.047 ^Bqr^	6.27 ± 0.012 ^Br^	6.42 ± 0.022 ^Bq^	6.40 ± 0.029 ^Bqr^	0.019	<0.001
Day 6	6.71 ± 0.014 ^Ap^	6.65 ± 0.085 ^Ap^	6.34 ± 0.064 ^Aq^	6.56 ± 0.074 ^Ap^	6.72 ± 0.066 ^Ap^	0.022	<0.001
SEM	0.021	0.019	0.013	0.015	0.014		
*p* value	<0.001	<0.001	<0.001	<0.001	<0.001		
TBARS (mg malondialdehyde/kg)							
Day 0	0.12 ± 0.006 ^C^	0.12 ± 0.002 ^C^	0.12 ± 0.002 ^C^	0.12 ± 0.003 ^C^	0.12 ± 0.004 ^C^	0.002	0.64
Day 3	0.22 ± 0.003 ^Bp^	0.21 ± 0.006 ^Bp^	0.19 ± 0.004 ^Bq^	0.22 ± 0.002 ^Bp^	0.22 ± 0.002 ^Bp^	0.002	<0.001
Day 6	0.36 ± 0.001 ^Ap^	0.35 ± 0.004 ^Aq^	0.33 ± 0.003 ^Ar^	0.35 ± 0.003 ^Aq^	0.35 ± 0.003 ^Aq^	0.002	<0.001
SEM	0.002	0.002	0.002	0.001	0.002		
*p* value	<0.001	<0.001	<0.001	<0.001	<0.001		
Peroxide value (meq O_2_/kg)							
Day 0	0.39 ± 0.031 ^C^	0.41 ± 0.025 ^B^	0.43 ± 0.042 ^B^	0.41 ± 0.036 ^B^	0.39 ± 0.038 ^B^	0.018	0.57
Day 3	1.55 ± 0.070 ^Bp^	1.35 ± 0.101 ^Apq^	0.95 ± 0.151 ^Ar^	1.28 ± 0.040 ^Aq^	1.01 ± 0.162 ^Ar^	0.067	<0.001
Day 6	1.68 ± 0.040 ^A^	1.25 ± 0.345 ^A^	1.07 ± 0.241 ^A^	1.27 ± 0.162 ^A^	1.25 ± 0.201 ^A^	0.128	0.066
SEM	0.026	0.119	0.097	0.058	0.089		
*p* value	<0.001	0.003	0.007	<0.001	0.001		
TVB-N (mg/kg)							
Day 0	78.9 ± 2.27 ^C^	79.1 ± 4.55 ^C^	78.8 ± 2.71 ^C^	78.5 ± 2.66 ^C^	79.1 ± 2.35 ^Cp^	1.01	0.99
Day 3	146 ± 1.50 ^Bp^	117 ± 2.12 ^Br^	88.1 ± 2.22 ^Bs^	137 ± 2.83 ^Bq^	120 ± 3.40 ^Br^	2.78	<0.001
Day 6	246 ± 2.91 ^Ap^	171 ± 1.68 ^As^	114 ± 1.75 ^At^	230 ± 2.25 ^Aq^	209 ± 10.3 ^Ar^	1.67	<0.001
SEM	0.77	1.02	0.75	0.86	2.14		
*p* value	<0.001	<0.001	<0.001	<0.001	<0.001		

Mean values bearing different superscripts (A, B, C) in a column and (p, q, r, s, t) in a row differ significantly (*p* < 0.05). Control: without any added Zn; ZnO: 20 mg/kg of inorganic ZnO; CZnO-NP: 20 mg/kg of chemically synthesized ZnO nanoparticles: ZnA: 20 mg/kg of inorganic zinc acetate: GZnO-NP: 20 mg/kg of green synthesized ZnO nanoparticles; TBARS: thiobarbituric acid reactive substance; TVB-N: total volatile base nitrogen.

**Table 2:** Effect of different zinc sources on the microbial (log10 cfu/g) loads (mean ± standard error) of minced fish during refrigerated storage (4 ± 1 °C).

Item	Treatment					SEM	*p* Value
	Control	ZnO	CZnO-NP	ZnA	GZnO-NP		
Total viable counts (log10 CFU/g)							
Day 0	4.03 ± 0.053 ^C^	4.01 ± 0.033 ^C^	4.01 ± 0.072 ^C^	4.01 ± 0.26 ^C^	4.03 ± 0.031 ^C^	0.016	0.90
Day 3	4.48 ± 0.025 ^Bp^	4.41 ± 0.015 ^Bq^	4.23 ± 0.029 ^Bs^	4.37 ± 0.023 ^Bq^	4.30 ± 0.026 ^Br^	0.008	<0.001
Day 6	5.54 ± 0.037 ^Ap^	5.31 ± 0.060 ^Aq^	4.82 ± 0.064 ^Ar^	5.31 ± 0.059 ^Aq^	5.29 ± 0.018 ^Aq^	0.017	<0.001
SEM	0.013	0.014	0.019	0.013	0.009		
*p* value	<0.001	<0.001	<0.001	<0.001	<0.001		
Coliforms (log10 CFU/g)							
Day 0	3.27 ± 0.055 ^C^	3.16 ± 0.127 ^C^	3.17 ± 0.159 ^B^	3.19 ± 0.161 ^C^	3.18 ± 0.151 ^B^	0.078	0.87
Day 3	3.72 ± 0.031 ^Bp^	3.56 ± 0.021 ^Bq^	3.12 ± 0.046 ^Bs^	3.52 ± 0.045 ^Bq^	3.28 ± 0.085 ^Br^	0.032	<0.001
Day 6	4.38 ± 0.187 ^Ap^	4.27 ± 0.117 ^Ap^	3.97 ± 0.092 ^Aq^	4.24 ± 0.140 ^Ap^	4.21 ± 0.061 ^Ap^	0.073	0.029
SEM	0.067	0.058	0.068	0.073	0.061		
*p* value	<0.001	<0.001	<0.001	<0.001	<0.001		
*Vibrio* spp. (log10 CFU/g)							
Day 0	2.42 ± 0.048 ^B^	2.42 ± 0.052 ^B^	2.41 ± 0.064 ^B^	2.42 ± 0.037 ^C^	2.42 ± 0.065 ^C^	0.018	0.97
Day 3	3.91 ± 0.018 ^Ap^	3.82 ± 0.053 ^Ap^	3.27 ± 0.054 ^As^	3.65 ± 0.033 ^Bq^	3.47 ± 0.042 ^Br^	0.014	<0.001
Day 6	4.01 + 0.047 ^Ap^	3.86 ± 0.032 ^Aq^	3.31 ± 0.014 ^As^	3.78 ± 0.074 ^Aqr^	3.66 ± 0.037 ^Ar^	0.015	<0.001
SEM	0.013	0.016	0.016	0.017	0.016		
*p* value	<0.001	<0.001	<0.001	<0.001	<0.001		
*Staphylococcus* (log10 CFU/g)							
Day 0	3.63 ± 0.028 ^C^	3.62 ± 0.044 ^C^	3.62 ± 0.046 ^C^	3.63 ± 0.050 ^C^	3.63 ± 0.043 ^C^	0.014	0.99
Day 3	4.24 ± 0.025 ^Bp^	3.98 ± 0.043 ^Bqr^	3.76 ± 0.024 ^Bs^	4.02 ± 0.035 ^Bq^	3.90 ± 0.020 ^Br^	0.010	<0.001
Day 6	5.15 ± 0.017 ^Ap^	5.05 ± 0.032 ^Apq^	4.50 ± 0.033 ^As^	5.04 ± 0.044 ^Aq^	4.65 ± 0.045 ^Ar^	0.011	<0.001
SEM	0.008	0.013	0.012	0.015	0.013		
*p* value	<0.001	<0.001	<0.001	<0.001	<0.001		

Mean values bearing different superscripts (A, B, C) in a column and (p, q, r, s) in a row differ significantly (*p* < 0.05). Control: without any added Zn; ZnO: 20 mg/kg of inorganic ZnO; CZnO-NP: 20 mg/kg of chemically synthesized ZnO nanoparticles: ZnA: 20 mg/kg of inorganic zinc acetate: GZnO-NP: 20 mg/kg of green synthesized ZnO nanoparticles.

**Table 3:** Effect of different zinc sources on sensory attributes (mean ± standard error) of minced fish during refrigerated storage (4 ± 1 °C).

Item	Treatment					SEM	*p* Value
	Control	ZnO	CZnO-NP	ZnA	GZnO-NP		
Color							
Day 0	4.83 ± 0.289 ^A^	4.70 ± 0.173 ^A^	4.76 ± 0.252 ^A^	4.77 ± 0.251 ^A^	4.83 ± 0.278 ^A^	0.147	0.96
Day 3	3.68 ± 0.161 ^B^	3.75 ± 0.250 ^B^	3.90 ± 0.361 ^B^	3.80 ± 0.264 ^B^	3.78 ± 0.683 ^B^	0.224	0.97
Day 6	2.67 ± 0.281 ^Cqr^	2.93 ± 0.115 ^Cpq^	3.25 ± 0.250 ^Cp^	2.85 ± 0.401 ^Cpqr^	2.33 ± 0.218 ^Cr^	0.165	0.030
SEM	0.146	0.108	0.169	0.183	0.265		
*p* value	<0.001	<0.001	0.002	0.001	0.002		
Odor							
Day 0	4.83 ± 0.278 ^A^	4.83 ± 0.255 ^A^	4.83 ± 0.268 ^A^	4.83 ± 0.240 ^A^	4.83 ± 0.296 ^A^	0.167	1.00
Day 3	2.90 ± 0.361 ^B^	3.42 ± 0.382 ^B^	3.47 ± 0.503 ^B^	3.52 ± 0.225 ^B^	3.62 ± 0.625 ^B^	0.254	0.37
Day 6	2.27 ± 0.208 ^Cqr^	2.97 ± 0.058 ^Cpq^	3.33 ± 0.288 ^Cp^	2.53 ± 0.757 ^Cqr^	2.00 ± 0.102 ^Cr^	0.218	0.010
SEM	0.169	0.161	0.216	0.280	0.232		
*p* value	<0.001	<0.001	0.005	0.003	<0.001		

Mean values bearing different superscripts (A, B, C) in a column and (p, q, r) in a row differ significantly (*p* < 0.05). Control: without any added Zn; ZnO: 20 mg/kg of inorganic ZnO; CZnO-NP: 20 mg/kg of chemically synthesized ZnO nanoparticles: ZnA: 20 mg/kg of inorganic zinc acetate: GZnO-NP: 20 mg/kg of green synthesized ZnO nanoparticles.

**Table 4:** Pearson (*r*) correlation among the variables in this study.

Variables ^1^	pH	TBARS	Peroxide Value	TVB-N	TVC	Coliforms	*Vibrio* spp.	*Staph* spp.	Color	Odor
pH	.	<0.01	<0.01	<0.01	<0.01	<0.01	<0.01	<0.01	<0.01	<0.01
TBARS	0.79	.	<0.01	<0.01	<0.01	<0.01	<0.01	<0.01	<0.01	<0.001
Peroxide value	0.84	0.80	.	<0.01	<0.01	<0.01	<0.01	<0.01	<0.01	<0.01
TVB-N	0.88	0.88	0.80	.	<0.01	<0.01	<0.01	<0.01	<0.01	<0.01
TVC	0.83	0.97	0.76	0.94	.	<0.01	<0.01	<0.01	<0.01	<0.01
Coliforms	0.84	0.95	0.76	0.92	0.98	.	<0.01	<0.01	<0.01	<0.01
*Vibrio* spp.	0.82	0.82	0.98	0.79	0.77	0.79	.	<0.01	<0.01	<0.01
*Staph* spp.	0.83	0.96	0.77	0.93	0.98	0.98	0.78	.	<0.01	<0.01
Color	−0.84	−0.98	−0.85	−0.90	−0.95	−0.92	−0.87	−0.92	.	<0.01
Odor	−0.87	−0.92	−0.92	−0.90	−0.89	−0.86	−0.92	−0.86	0.97	.

^1^ Values in the bottom half of the table are for correlations, and values in the top half of the table are for *p* values. TBARS: thiobarbituric acid reactive substance; TVB-N: total volatile base nitrogen; TVC: total viable counts; *Staph*: *Staphylococcus*.

## Data Availability

The original contributions presented in the study are included in the article/Supplementary Material, further inquiries can be directed to the corresponding authors.

## Supplementary Materials

The following supporting information can be downloaded at: https://www.mdpi.com/article/10.3390/foods13172810/s1, Figure S1: Effect of chemically synthesized ZnO-NPs on color of minced fish at different days. Data points bearing different letters (a, b, c, d) differ significantly (*p* < 0.05) within a day.
