# Supplementary material for: Effects of Chemically and Green Synthesized Zinc Oxide Nanoparticles on Shelf Life and Sensory Quality of Minced Fish (Pangasius hypophthalmus)
Source: Foods. 2024 Sep 4;13(17):2810. doi: 10.3390/foods13172810 (PMC11394675; doi:10.3390/foods13172810)
Supplement: Supplementary file 1 [file foods-13-02810-s001.zip › foods-3166510-supplementary.pdf]

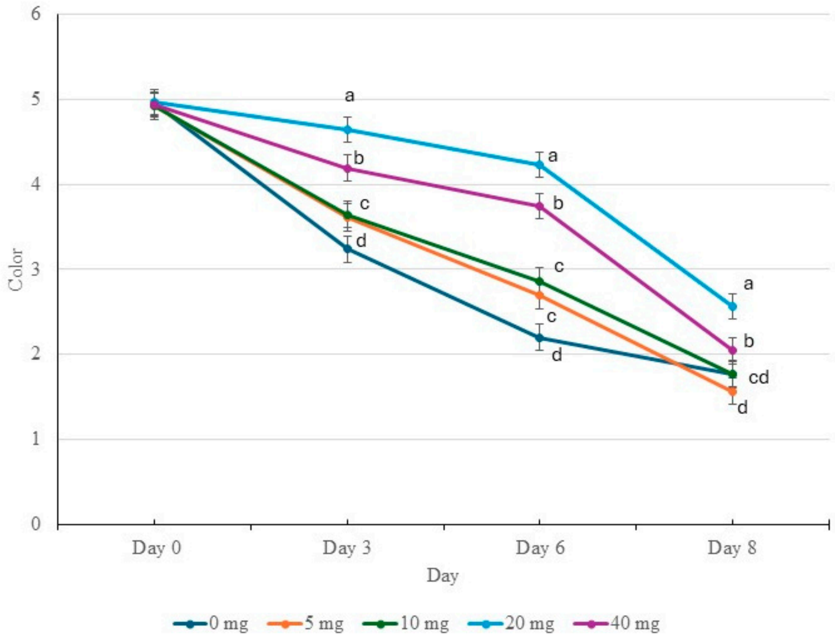

Supplementary Figure S1. Effect of chemically synthesized ZnO-NPs on color of minced fish at different days. Data points bearing different letters (a,b,c,d) differ significantly ($P<0.05$) within a day.
